# Total impact of oxidative stress genes on cardiovascular events—a 7-year follow-up study

**DOI:** 10.1007/s13353-022-00741-9

**Published:** 2023-01-23

**Authors:** Milena Racis, Anna Stanisławska-Sachadyn, Wojciech Sobiczewski, Marcin Wirtwein, Michał Krzemiński, Andrzej Rynkiewicz, Bartosz Wasąg, Miłosz Jaguszewski, Marcin Gruchała

**Affiliations:** 1 First Department of Cardiology, Medical University of Gdansk, Ul. Smoluchowskiego 17, 80-214 Gdańsk, Poland; 2 Department of Molecular Biotechnology and Microbiology, Gdansk University of Technology, Ul. Narutowicza 11/12, 80-233 Gdańsk, Poland; 3 Department of Biology and Genetics, Medical University of Gdansk, Ul. Dębinki 1, 80-211 Gdańsk, Poland; 4 Department of Pharmacology, Medical University of Gdansk, Ul. Dębowa 23, 80-204 Gdańsk, Poland; 5 Institute of Applied Mathematics, Faculty of Applied Physics and Mathematics, Gdansk University of Technology, 80-233 Gdańsk, Poland; 6 Department of Cardiology and Cardiosurgery, University of Warmia and Mazury in Olsztyn, Al. Warszawska 30, 10-082 Olsztyn, Poland

**Keywords:** Gene polymorphism, SNP, GRS, Oxidative stress, Cardiovascular disease, CV events

## Abstract

Cardiovascular (CV) events are the number one cause of lifetime disability and deaths worldwide. It is well known that traditional risk factors do not fully correlate with clinical outcomes; therefore, searching for other markers that would explain CV events’ occurrence seems essential. Of importance, one of the main factors at the origin of CV events is oxidative stress, causing inflammation and atherosclerotic plaque instability. Therefore, the present study was conducted to evaluate eight carefully selected genetic polymorphisms related to oxidative stress as risk modifiers for CV events. A cohort of 1020 patients with coronary atherosclerosis was analysed in a 7-year follow-up observational study. The following end points were assessed: CV death, myocardial infarction (MI) and a combined end point of CV death/MI/stroke. Our results show that single polymorphisms are not significant cardiovascular disease risk factors, but genetic risk score (GRS), defined as the accumulation of our eight studied polymorphisms, was significantly associated with the three. Specifically, low GRS was associated with a higher risk of CV death, MI and CV death/MI/stroke. In conclusion, when regarding CV events, GRS investigated here can become clinically meaningful and undoubtedly adds to the knowledge in stratifying the risk of CV events.

**Supplementary Information:**

The online version contains supplementary material available at 10.1007/s13353-022-00741-9.

## Introduction

Despite increasingly intensive primary and secondary prevention, cardiovascular (CV) events continue to be the world’s leading cause of death (Virani et al. 2020), typically occurring due to a sudden atherosclerotic plaque rupture in the presence of systemic inflammation (d’Alessandro et al. 2020; Alfaddagh et al. 2020). Traditional risk factors do not fully correlate with clinical outcomes; therefore, searching for other markers that explain the different courses of cardiovascular disease (CVD) seems rational (Daiber et al. 2021; Lechner et al. 2020; Pavkova Goldbergova et al. 2017). As oxidative stress, identified as an increase of reactive oxygen species (ROS), leads to oxidative damage in cells and promotes inflammation, it may be perceived as an essential player in the disruption and thrombosis of atherosclerotic plaques (Sies 2015; Kibel et al. 2020). Moreover, as clinical data suggest, CVD has a strong genetic component. Based on these two facts, we presumed that selected single nucleotide polymorphisms (SNPs) within genes encoding crucial enzymes of redox regulation and the accumulation of those SNPs could give new insights into the molecular basis of CVD (Katakami et al. 2014; Augusciak-Duma et al. 2021).

In the present 7-year follow-up observation study, we used eight selected single nucleotide polymorphisms (SNPs) within genes encoding crucial enzymes of redox regulation and the accumulation of those variants defined as genetic risk score (GRS), under the assumption that they may impact CV events in a significant manner. Specifically, those eight SNPs and the GRS based on the accumulation of the pro-atherosclerotic variants were assessed as the potential risk modifiers for CV death, nonfatal myocardial infarction (MI) and the combined end point of cardiovascular death, nonfatal MI or nonfatal cerebral stroke (CV death/MI/stroke). Our secondary aim was to examine whether the genetic risk factors for atherosclerosis are simultaneously risk factors for CV events.

All the selected variants are discussed as functional. Briefly, paraoxonase 1 (PON1) is involved in LDL oxidation and oxidized lipid degradation. The *PON1* c. 575G allele has been linked to higher paraoxonase activity (Humbert et al. 1993) and has been described as a cardiovascular disease risk allele (Roest et al. 2007; Liu et al. 2014). Myeloperoxidase (MPO) is involved in LDL oxidation and ROS production within atherosclerotic plaques (Daugherty et al. 1994). Manganese superoxide dismutase (MnSOD, the *SOD2* gene) is an enzyme catalysing the conversion of reactive superoxide radicals to hydrogen peroxide (Lian et al. 2019). The *SOD2* c.47TT genotype has been associated with increased CHD risk (Jones et al. 2010). Glutamate-cysteine ligase (GCL, *GCLM* gene) synthesizes the primary intracellular antioxidant, glutathione (Franklin et al. 2009), while the *GCLM* c.-590 T allele has been associated with lower plasma glutathione levels (Katakami et al. 2009). Endothelial nitric oxide synthase (eNOS) produces nitric oxide reported to inhibit lipid peroxidation, relaxes smooth muscles and increase blood flow (Yetik-Anacak and Catravas 2006). The eNOS c.894 T allele (rs1799983) has been linked to reduced promoter activity and diminished nitric oxide production (Oliveira-Paula et al. 2016). NADPH oxidase, the regulatory subunit of which is encoded by the *CYBA* gene, is involved in ROS generation. The *CYBA* c.214 T allele has been described as an atherosclerosis and brain stroke risk factor (Inoue et al. 1998), while c.214C allele has been linked to diminished ROS production (Bedard et al. 2009). Another variant in the *CYBA* promoter region c.-932G has been linked to increased p22Phox expression and ROS production (San José et al. 2008).

The choice of the pro-atherosclerotic variant in each locus was based on the results of studies referenced in Table 1 and the results of our research (Racis et al. 2020b), where those variants were studied in association with the extent of coronary atherosclerosis.

**Table 1:** Baseline characteristics of the investigated SNPs (single nucleotide polymorphisms)

Enzyme	Gene	SNP	Ref	Gene location	Phenotypic consequence of variant	rs	MAF*
Paraoxonase1 (PON1)	*PON1*	*PON1* c.575 A > G	Shunmoogam et al. (2018), Deng et al. (2020)	7q21.3; exon 6	^192^Gln → Arg; higher PON1 activity	rs662	*G* = 0.315
Myeloperoxidase (MPO)	*MPO*	*MPO* c.-463 A > G	Daugherty et al. (1994), Ergen et al. (2011), Racis et al. (2020a)	17q23.1; promoter	Increased gene expression	rs2333227	*A* = 0.088
Manganese superoxide dismutase (MnSOD)	*SOD2*	*SOD2* c.47C > T	Bresciani et al. (2013), Lian et al. (2019)	6q25.3; exon 2	^16^Val → Ala	rs4880	*G* = 0.489
γ-Glutamyl-cysteine ligase (GCL)	*GCLM*	*GCLM* c.588 C > T	Nakamura et al. (2002), Franklin et al. (2009)	1p22.1; promoter	Decreased glutathione production	rs41303970	*T* = 0.178
Endothelial nitric oxide synthase (eNOS)	*NOS3*	* NOS3* c.894 G > T	Aimo et al. (2019), Oliveira-Paula et al. (2016), Erbs et al. (2006)	7q36; exon 7	^298^Glu → Asp; lower NO and higher lipid levels	rs1799983	*T* = 0.315
		* NOS3* c.-786 C > T		7q36; promoter	Increased gene expression by 50%	rs2070744	*C* = 0.349
NADPH oxidase (p22phox subunit)	*CYBA*	*CYBA* c.214 T > C	Inoue et al. (1998), San José et al. (2008), Racis et al. (2020a)	16q24; exon 4	^72^His → Tyr; affected heme binding	rs4673	*T* = 0.342
		*CYBA* c.-932 G > A		16q24; promoter	Decreased gene expression	rs9932581	*A* = 0.409

*The frequencies of MAF are based on the ALFA project

## Materials and methods

### Study population

**Fig. 1:**
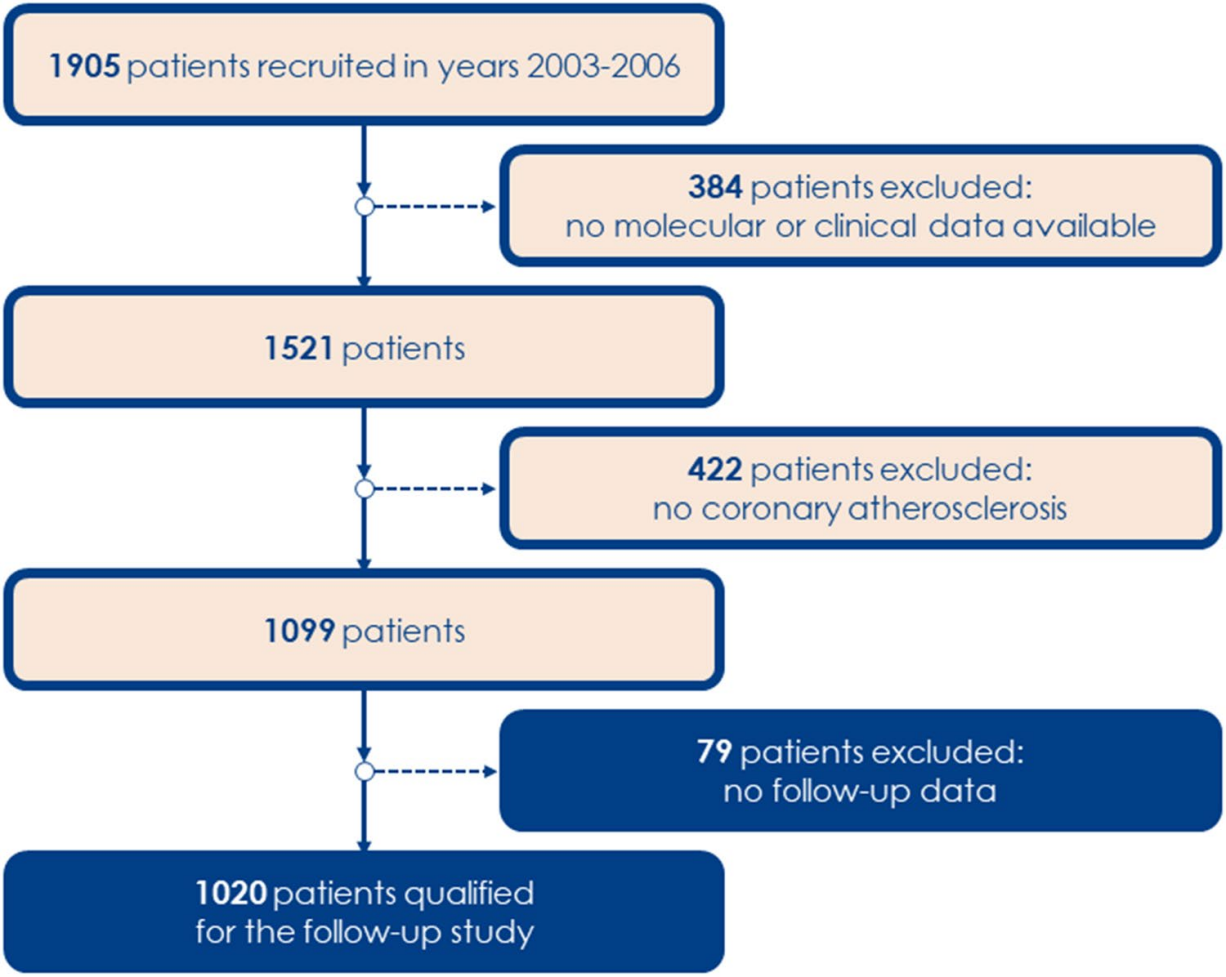
The inclusion and exclusion criteria of the patients recruited into the study

Health-related data from 1905 patients referred for diagnostic coronary angiography to the First Department of Cardiology of the Medical University of Gdansk between 2003 and 2006 were used to compile a study population (Wirtwein et al. 2017; Wirtwein et al. 2018). In this project, as in our previous publication (Racis et al. 2020b), the same group with coronary atherosclerosis confirmed in coronary angiography was enrolled; however, the final analysis was conducted on 1020 individuals since 79 patients lacked follow-up data; detailed inclusion and exclusion criteria are presented in Fig. 1.

### Follow-up study

The prospective data were obtained from the National Polish Health Service by means of the patients’ names and Polish residence identification numbers. All patients were observed from the date of coronary angiography until 31 December 2011. The data used to determine the evaluated end points were collected in a 7-year follow-up (mean, 96 months).

The end points, also defined as CV events, were as follows: (1) CV death, (2) nonfatal myocardial infarction (MI) and (3) a combined end point of CV death, nonfatal MI or nonfatal cerebral stroke (CV death/MI/stroke). The International Statistical Classification of Diseases and Related Health Problems defined the term CV death. The term MI was applied to non-ST-elevation MI and ST-elevation MI. MI and stroke diagnoses were performed according to the European Society of Cardiology and the European Stroke Organization guidelines, respectively.

### Genetic risk score

To create genetic risk score (GRS), we selected eight single-nucleotide polymorphisms (SNPs), as mentioned above. Selection criteria included the following: (1) SNP has been described as functional in previous studies, and (2) minor allele frequency was > 0.05. The analysis included the following SNPs: *PON1* c.575A > G, *MPO* c.-463G > A, *SOD2* c.47 T > C, *GCLM* c.-590C > T, *NOS3* c.894G > T, *NOS3* c.-786 T > C, *CYBA* c.214C > T and *CYBA* c.-932A > G; these SNPs constituted the same set of SNPs analysed in our previous study. Primers, probe sequences, concentrations of reagents and genotyping conditions are listed in Table S1. The risk alleles were defined according to their potential to increase the extent of atherosclerosis, as presented in our previous publication (Racis et al. 2020b). After each SNP was investigated individually, the additive effect of the eight SNPs was analysed as a GRS that reflected the total impact of genetic variants.

**Fig. 2:**
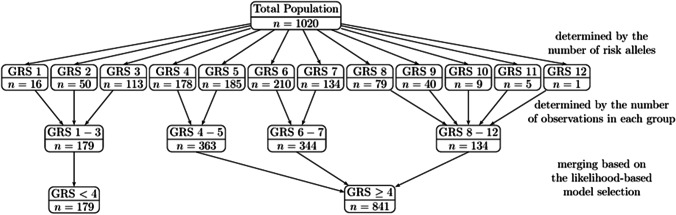
Distribution of risk alleles and GRS models

To construct the GRS model, the number of risk alleles in each genetic locus was established (0: no risk allele; 1: one risk allele; 2: two risk alleles) and then results from all eight loci of each patient were summarized. Although seventeen groups of patients could be created (from 0 [having no risk alleles] to 16 [having risk alleles exclusively]), only 12 groups were selected—from the group with one to the group with 12 risk alleles—because no patients had 0, 13, 14, 15 or 16 risk alleles present. Thus, patients were divided into the following groups: 1 (*n* = 16), 2 (*n* = 50), 3 (*n* = 113), 4 (*n* = 178), 5 (*n* = 185), 6 (*n* = 210), 7 (*n* = 134), 8 (*n* = 79), 9 (*n* = 40), 10 (*n* = 9), 11 (*n* = 5) and 12 (*n* = 1). Then, these 12 groups were merged into four GRS groups according to the number of patients: the first three groups (carriers of 1, 2 and 3 alleles [*n* = 179]); the last five groups (carriers of 8–12 alleles [*n* = 136]) and the middle groups in sets of two (carriers of 4 and 5 [*n* = 363]; 6 and 7 alleles [*n* = 344]). Ultimately, according to the analysis of the Kaplan-Meyer curves, which reflected the different behaviour of groups 1–3, based on the likelihood adaptive fusing model selection, the whole population was divided into two final groups—GRS < 4 (*n* = 179) and GRS ≥ 4 (*n* = 843), which were named the low GRS group and the high GRS group, respectively (Fig. 2).

### Statistical analysis

Continuous variables were expressed as means ± standard deviations (SDs). Categorical (dichotomous) variables were expressed as frequencies (%). For all SNPs, the risk allele frequencies were calculated. Hazard ratios (HRs) and 95% CIs were determined for each event using multivariate Cox proportional hazards models, in which one key independent variable (risk allele, GRS) was adjusted for age and sex. The clinical characteristics of the patients were presented as means ± SDs and as medians for continuous variables (i.e. body mass index, triglyceride level) or percentages for categorical variables (i.e. smoking history, hypertension, diabetes). Smoking status was self-reported. Deviations from the Hardy–Weinberg equilibrium for the genotypes were assessed using a chi-square test. The chi-square tests compared the frequencies of categorical variables between groups, and the Wilcoxon test compared levels of continuous variables between groups. The follow-up events were presented using the Kaplan–Meier curves, and the odds ratio (OR) and the log-rank test assessed the differences in survival (cumulative incidence of events) among different groups. The model with two groups according to the number of risk alleles (GRS < 4 and GRS ≥ 4) was chosen after adaptive fusing based on the likelihood ratio method as the one with the highest log-likelihood (Sitko and Biecek 2017). An alpha value of 0.05 was considered significant. All statistical analyses were performed using R version 4.0.2 (https://www.R-project.org).

## Results

### Baseline characteristics

The characteristics of the entire population and of the two GRS subgroups are presented in Table 2. There were no statistically significant differences between the two GRS subgroups regarding the clinical characteristics and the prevalence of risk factors. All the SNPs were in Hardy–Weinberg equilibrium. During the median 7-year follow-up, 47 incidences of CV death, 82 cases of nonfatal MI and 36 cases of nonfatal cerebral stroke were reported. The prevalence of the combined end point (CV death/MI/stroke) was 162 cases.

**Table 2:** Baseline subject characteristics

Parameters	Total *n* = 1020	GRS < 4 *n* = 179	GRS ≥ 4 *n* = 841	*p*-value
Age (years): mean ± SD	64.1 ± 9.4	63.5 ± 8.9	64.3 ± 9.5	*ns*
Gender (male): *n* (%)	676 (66.0%)	123 (69%)	553 (66%)	*ns*
BMI (kg/m^2^): mean ± SD	28.0 ± 4.1	27.9 ± 4.1	28.1 ± 4.1	*ns*
Hypertension: *n* (%)	796 (81.1%)	143 (83%)	653 (81%)	*ns*
Diabetes: *n* (%)	243 (25.0%)	52 (30%)	191 (24%)	*ns*
HbA1c, mean ± SD	6.32 ± 1.16	6.36 ± 1.16	6.32 ± 1.16	*ns*
Total cholesterol (mg/dL), mean ± SD	206.6 ± 51.6	206.2 ± 46.2	206.7 ± 52.0	*ns*
LDL cholesterol (mg/dL), mean ± SD	123.3 ± 43.8	125.0 ± 43.1	122.9 ± 43.9	*ns*
HDL cholesterol (mg/dL), mean ± SD	54.5 ± 13.9	53.9 ± 14.8	54.7 ± 13.7	*ns*
Triglycerides (mg/dL), mean ± SD	146.5 ± 92.7	142.2 ± 64.9	147.4 ± 97.9	*ns*
History of smoking: *n* (%)	632 (67.0%)	115 (69%)	517 (67%)	*ns*
CVD in family: *n* (%)	546 (54.0%)	85 (51%)	421 (55%)	*ns*

*ns*, nonstatistically significant; *n*, number of subjects included into analysis; *SD*, standard deviation; *BMI*, body mass index; *LDL*, low-density lipoprotein; *HDL*, high-density lipoprotein; *CVD*, cardiovascular disease.

### Individual SNPs and CV events

The relationship between individual SNP and the risk of CV events was evaluated. Cox proportional hazard regression analyses accounting for age, sex and genotype showed the clinical significance of the *CYBA* c.214C > T SNP, in which the T allele was protective against CV death (HR = 0.59; 95% CI: 0.37–0.94; log-rank *p* = 0.026). In case of analyses involving other SNPs, no statistically significant outcomes with follow-up end points were observed (Table 3).

**Table 3:** Hazard ratio of CV death, MI and CV death/MI/stroke regarding individual SNPs and GRS models

SNP	Risk allele	CV death			MI			CV death/MI/stroke		
		HR	95% CI	*p*-value	HR	95% CI	*p*-value	HR	95% CI	*p*-value
*PON1* c.575 A > G	G	0.82	0.51–1.34	*ns*	1.06	0.75–1.51	*ns*	0.97	0.75–1.24	*ns*
*MPO* c.-463 A > G	A	1.14	0.67–1.93	*ns*	0.70	0.44–1.12	*ns*	0.93	0.69–1.26	*ns*
*SOD2* c.47C > T	T	0.74	0.48–1.12	*ns*	1.16	0.85–1.58	*ns*	0.96	0.77–1.20	*ns*
*GCLM* c.588 C > T	T	0.68	0.42–1.10	*ns*	1.28	0.82–1.98	*ns*	0.95	0.72–1.25	*ns*
*NOS3* c.894 G > T	T	0.65	0.39–1.08	*ns*	0.88	0.62–1.25	*ns*	0.79	0.61–1.03	*ns*
* NOS3* c.-786 C > T	C	0.86	0.57–1.31	*ns*	0.79	0.57–1.09	*ns*	0.81	0.65–1.02	*ns*
*CYBA* c.214 T > C	T	0.59	0.37–0.94	**0.026**	0.84	0.60–1.16	*ns*	0.91	0.73–1.15	*ns*
*CYBA* c.-932 G > A	G	1.01	0.67–1.52	*ns*	0.93	0.68–1.27	*ns*	0.92	0.73–1.14	*ns*
GRS models										
GRS^u^		0.87	0.75–1.01	*ns*	0.91	0.81–1.02	*ns*	0.92	0.85–0.99	**0.037**
GRS^a^		0.87	0.74–1.01	*ns*	0.91	0.81–1.02	*ns*	0.92	0.85–1.00	**0.043**
GRS ≥ 4^u^		0.49	0.26–0.92	**0.027**	0.54	0.33–0.87	**0.011**	0.60	0.42–0.85	**0.004**
GRS ≥ 4^a^		0.47	0.25–0.89	**0.020**	0.52	0.33–0.87	**0.011**	0.59	0.42–0.84	**0.003**

Bold indicate significant results

*ns*, nonstatistically significant; *SNP*, single nucleotide polymorphism; *CV* death, cardiovascular death; *MI*, myocardial infarction; *HR*, hazard ratio; *95% CI*, 95% confidence interval; *GRS*, genetic risk score; Cox proportional hazard regression analyses: ^u^unadjusted; ^a^adjusted for age and sex.

### GRS and CV events

**Fig. 3:**
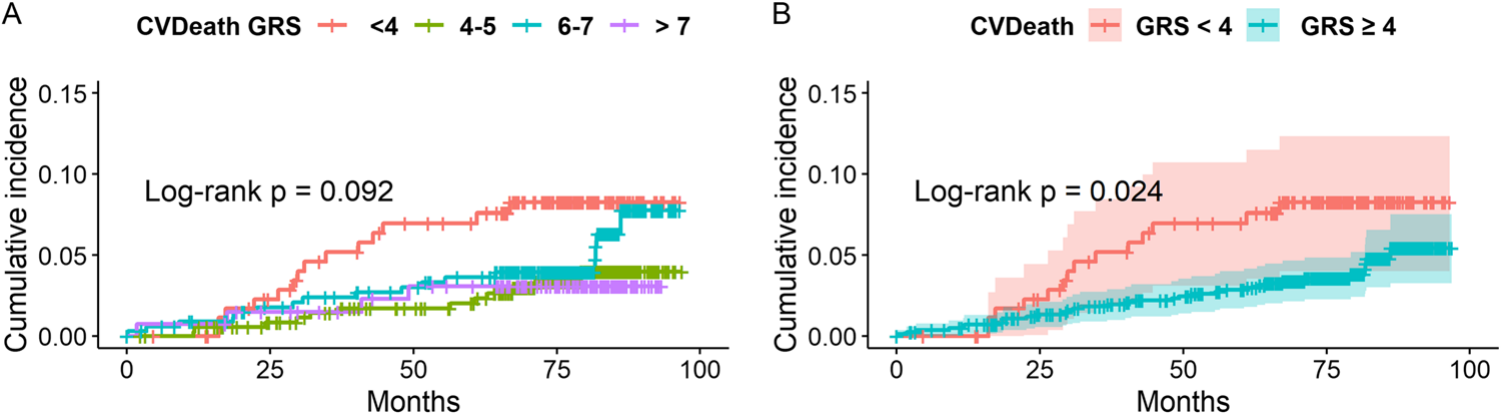
**A** The cumulative probability of CV death in patients with < 4, 4–5, 6–7 and ≥ 8 risk alleles. There was a trend that reveals differences in CV death rate between the GRS groups (*p *= 0.092). **B **The cumulative probability of CV death in patients with < 4 (*n *= 179) and ≥ 4 risk alleles (*n *= 843). The risk of CV death was significantly lower in the group with ≥ 4 atherosclerosis risk alleles (*p *= 0.024). 7-year Kaplan-Meyer curves of probability of CV death (95% Hall-Wellner bands (B)). A log-rank test was used to evaluate the difference in MI incidence between patients with various GRS

**Fig. 4:**
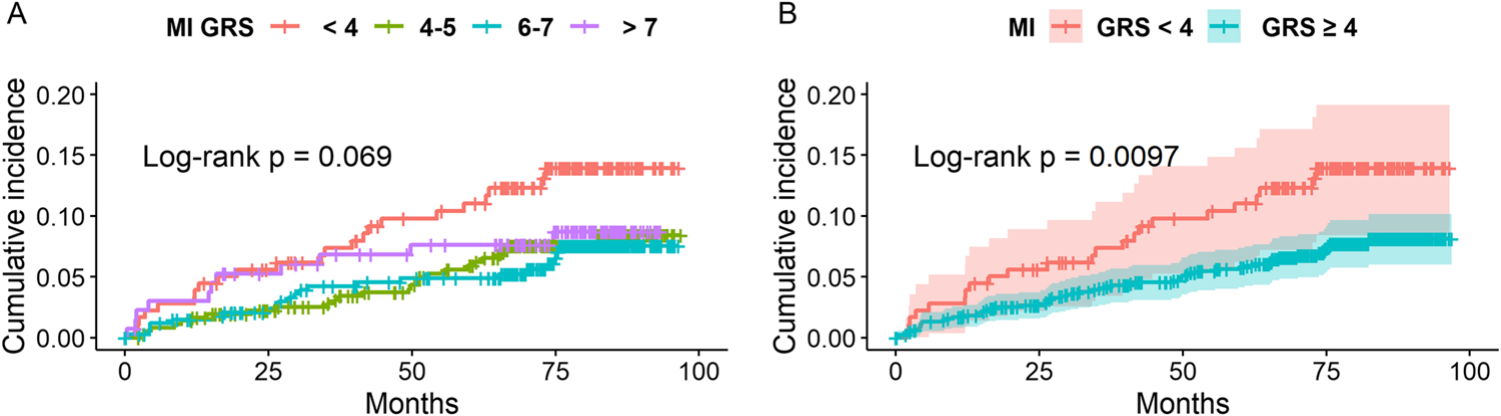
**A** The cumulative probability of MI in patients with < 4, 4–5, 6–7 and ≥ 8 risk alleles. There was a trend that reveals differences in MI rate between the GRS groups (*p *= 0.069). **B** The cumulative probability of MI in patients with < 4 (*n *= 179) and ≥ 4 risk alleles (*n *=843). The risk of MI was significantly lower in the group with ≥ 4 atherosclerosis risk alleles (*p *= 0.0097). 7-year Kaplan-Meyer curves of probability of MI (95% Hall-Wellner bands (B)). A log-rank test was used to evaluate the difference in MI incidence between patients with various GRS

**Fig. 5:**
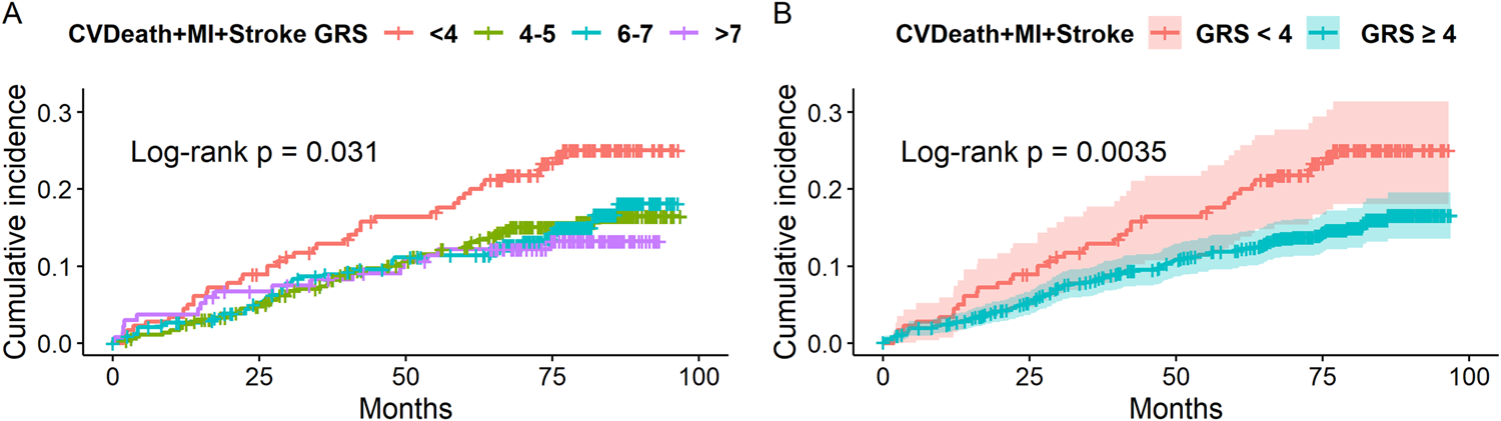
**A** The [DCJR1] cumulative probability of CVdeath/MI/stroke in patients with < 4, 4–5, 6–7 and ≥ 8 risk alleles. There was a statistically significant difference in CVdeath/MI/stroke rate between the GRS groups (*p *= 0.031). **B **The cumulative probability of CVdeath/MI/stroke in patients with < 4 (*n *= 179) and ≥ 4 risk alleles (*n *= 843). The risk of CVdeath/MI/stroke was significantly lower in the group with ≥ 4 atherosclerosis risk alleles (*p *= 0.0035). 7-year Kaplan-Meyer curves of probability of CVdeath/MI/stroke (95% Hall-Wellner bands (B)). A log-rank test was used to evaluate the difference in CVdeath/MI/stroke incidence between patients with various GRS

Among the patients with complete genetic data, the additive effect of eight SNPs on the studied follow-up end points was evaluated among patients in four GRS groups, depending on the number of risk alleles they carried: GRS 1–3 (*n* = 179), GRS 4–5 (*n* = 363), GRS 6–7 (*n* = 344) and GRS 8–12 (*n* = 136). The Kaplan–Meier curves depicting the cumulative probability of the follow-up end points revealed statistically significant differences in cumulative incidence of CV death/MI/stroke (log-rank *p* = 0.031) and trends regarding CV death and MI (log-rank *p* = 0.092 and *p* = 0.069, respectively) (Figs. 3a, 4a and 5a). After implementation of adaptive fusing based on the likelihood ratio method, the Kaplan–Meier curves of two final groups depicting the cumulative probability of the follow-up end points revealed a significantly lower risk of CV death (log-rank *p* = 0.024), nonfatal MI (log-rank *p* = 0.0097) and CV death/MI/stroke (log-rank *p* = 0.0035) in the high GRS group compared with the low GRS group (Figs. 3b, 4b and 5b).

Simultaneously, the Cox proportional hazard regression analyses revealed that the risk of CV death non-fatal MI and CV death/MI/stroke in the high GRS group was significantly lower than that in the low GRS group (HR = 0.49 [95% CI: 0.26–0.92; *p* = 0.027]; 0.54 [95% CI: 0.33–0.87; *p* = 0.011]; and 0.60 [95% CI: 0.42–0.85; *p* = 0.004], respectively). The outcome remained significant when adjusted for sex and age (HR = 0.47 [95% CI: 0.25–0.89; *p* = 0.020]; 0.52 [95% CI: 0.33–0.87; *p* = 0.011]; and 0.59 [95% CI: 0.42–0.84; *p* = 0.003], respectively) (Table 3). Furthermore, the OR analyses showed that the risk of CV events was significantly lower in the high GRS group than in the low GRS group (CV death OR = 0.56 [95% CI: 0.36–0.85; *p* = 0.006]; MI OR = 0.48 [95% CI: 0.25–0.92; *p* = 0.024]; and CV death/MI/stroke OR = 0.54 [95% CI: 0.37–0.81; *p* = 0.002]).

Our study results indicate that only one SNP—*CYBA c*.214T—was associated with one end point (CV death/MI/stroke). However, GRS was significantly associated with all three follow-up end points. According to these results, having fewer than four risk alleles within the eight investigated SNPs can be a risk factor for CV death, nonfatal MI and CVdeath/MI/stroke. Conversely, having four or more risk alleles within the eight investigated SNPs can be a protective factor against CV death, nonfatal MI and CVdeath/MI/stroke.

## Discussion

Oxidative stress is one of the main factors at the origin of CV events, which are the number one cause of lifetime disabilities and deaths worldwide. It is identified as an increase of reactive oxygen species (ROS), leading to oxidative damage in cells and promoting inflammation. Thus, it may be perceived as an essential player in the disruption of an atherosclerotic plaque showing adverse events. In the study, we investigated eight carefully chosen functional SNPs within genes encoding crucial enzymes of redox regulation and their additive effect on CV events’ occurrence. Our working hypothesis was that the accumulation of several variants, represented by GRS, influences redox status within the organism and, in this way, has an influence on CVD outcome.

Our results show that GRS is significantly associated with three studied follow-up end points: CV death, nonfatal MI and CVdeath/MI/stroke. It must be highlighted that one SNP may not be significantly associated with CVD manifestation; however, the accumulation of several genetic variants might act as risk modifiers for CV events.

Interestingly, we previously published data that analysed the impact of the same eight oxidative stress–related SNPs presented as GRS on the extent of atherosclerosis detected in coronary angiography (Racis et al. 2020b). According to our previous results, the high GRS, which makes CV events less frequent, was associated with more advanced coronary atherosclerosis. Thus, when interpreted together, both our studies showed the opposite trend: the genetic factors that favour atherosclerosis formation simultaneously make CV events less frequent. What may appear conflicting seems to be supported by clinical evidence—extensive atherosclerosis often does not result in an acute manifestation of cardiovascular disease. Conversely, individuals suffering from CV events often have not previously developed advanced atherosclerosis. Moreover, it seems to be in accordance with pathophysiological findings. Specifically, in 1985, it was determined that CV events are not usually the result of a slow-growing plaque that gradually affects the coronary artery lumen; rather, they are a result of a sudden plaque rupture from a plaque that was unstable before the incident although the artery was not critically narrowed (Sies and Cadenas 1985).

The response to the question of whether the high GRS built of pro-atherosclerotic alleles in the oxidative stress–related genes can impact the anatomy of the atherosclerotic plaque by making it more stable remains open. We may hypothesize that, although ROS are mainly associated with the clinical presentation of the disease, their biological significance as signalling molecules must also be appreciated since they are fundamentally important for the physiological regulation of biological activities (Dworakowski et al. 2006; Sies and Jones 2020; Brandes et al. 2018). Moreover, concerning molecular processes ongoing in mitochondria, moderately elevated levels of ROS can improve the systemic defence by inducing an adaptive, health-supporting response (Ristow and Zarse 2010; Ursini et al. 2016).

Our study has some limitations. Although we associate our specific GRS with clinical outcomes, the conclusions about how it influences oxidative stress remain uninvestigated, as we did not perform any functional studies on those eight SNPs. Another limitation of our study is that the GRS is based on only eight specific oxidative stress–related SNPs. Although those SNPs were carefully selected based on their importance in redox state balance in terms of their functionality, future studies with a wider range of oxidative stress–related factors would be of interest.

## Conclusions

According to our study, the investigated eight oxidative stress–related polymorphisms, *PON1* c.575A > G, *MPO* c.-463G > A, *SOD2* c.47 T > C, *GCLM* c.-590C > T, *NOS3* c.894G > T, *NOS3* c.-786 T > C, *CYBA* c.214C > T and *CYBA* c.-932A > G, are significantly associated with CV events’ occurrence. Specifically, the high GRS group containing the carriers of four or more alleles, which were described as pro-atherosclerotic, is associated with lower risk of CV death, nonfatal MI and combined risk of CV death/MI/stroke.

As a conclusion, our findings may indicate better routes in the genetic approach to clinical management of acute manifestation of cardiovascular disease.

## Supplementary Information

Below is the link to the electronic supplementary material.

Supplementary file1 (DOCX 15 KB)

## Data Availability

All primary data are available from the corresponding author upon the reasonable request of qualified researchers.
