# Supplementary material for: Total impact of oxidative stress genes on cardiovascular events—a 7-year follow-up study
Source: J Appl Genet. 2023 Jan 23;64(2):319–27. doi: 10.1007/s13353-022-00741-9 (PMC10076362; doi:10.1007/s13353-022-00741-9)
Supplement: Supplementary file 1 — Supplementary file1 (DOCX 15 KB) [file 13353_2022_741_MOESM1_ESM.docx]

| **SNVs** | **Probes, primers, other reagents** | | **Thermal profile** |
| --- | --- | --- | --- |
| **Taqman method** | | | |
| *PON1* c.575A>G  rs662 | A: FAM- CTACTTACAATCCTGGGAGATGT -BHQ1 | 50 nM | 95 °C for 10 min  95 °C for 30 s  55 °C for 30 s  repeat 60 times |
|  | G: HEX- TACTTACGATCCTGGGAGATGTA - BHQ1 | 50 nM |  |
|  | F: 5’- GTTGCTGTGGGACCTGA –3’ | 0.5µM |  |
|  | R: 5’- AGACAACATACGACCACG -3’ | 0.5µM |  |
| *MPO* c.-463G>A  rs2333227 | G: FAM- CTGAGGCGGGTGGATCACTTGA -BHQ1 | 50 nM | 95 °C for 10 min  95 °C for 30 s  64 °C for 30 s  repeat 60 times |
|  | A: HEX- CTGAGGCAGGTGGATCACTTGAG - BHQ1 | 50 nM |  |
|  | F: 5’- CACACAATGGTGAGCTGAGA –3’ | 0.5µM |  |
|  | R: 5’- TTTGTATTTTTCCTTAGGCAAGAAGC -3’ | 0.5µM |  |
| *GCLM* c.-590C>T  rs41303970 | C: FAM- CTGAACGCCGGGAGACCTCACCA -BHQ1 | 50 nM | 95 °C for 10 min  95 °C for 30 s  64 °C for 30 s  repeat 60 times |
|  | T: HEX- CCTGAACGCTGGGAGACCTCACCA - BHQ1 | 50 nM |  |
|  | F: 5’- CAGGCTGCCCTTTAAAGAG –3’ | 0.5µM |  |
|  | R: 5’- CCGCCTGGTGAGGTAGA -3’ | 0.5µM |  |
| *SOD2* c.47T>C  rs4880 | T: FAM- CTGGCTCCGGTTTTGGGGTATCT -BHQ1 | 50 nM | 95 °C for 10 min  95 °C for 30 s  62 °C for 30 s  repeat 60 times |
|  | C: HEX- TGGCTCCGGCTTTGGGGTATC - BHQ1 | 50 nM |  |
|  | F: 5’- CTTTCTCGTCTTCAGCACCA –3’ | 0.5µM |  |
|  | R: 5’- CGCGTTGATGTGAGGTTC -3’ | 0.5µM |  |
| *NOS3* c.894T>G  rs1799983 | T: FAM- CCAGATGATCCCCCAGAACTCTTCC -BHQ1 | 50 nM | 95 °C for 10 min  95 °C for 15 s  64 °C for 15 s  repeat 60 times |
|  | G: HEX- CAGATGAGCCCCCAGAACTCTTCCT - BHQ1 | 50 nM |  |
|  | F: 5’-CATTCAGCACGGCTGGA –3’ | 0.5µM |  |
|  | R: 5’- ACCTCAAGGACCAGCTC -3’ | 0.5µM |  |
| *NOS3* c.-786T>C  rs2070744 | C: FAM- TCCCTGGCCGGCTGACCCTGCCTCA -BHQ1 | 50 nM | 95 °C for 10 min  95 °C for 20 s  68 °C for 20 s  repeat 60 times |
|  | T: HEX- CTTCCCTGGCTGGCTGACCCTGCCTC - BHQ1 | 50 nM |  |
|  | F: 5’- TCCCACCAGGGCATCAA –3’ | 0.5µM |  |
|  | R: 5’- CCCTGTCATTCAGTGACG -3’ | 0.5µM |  |
| *CYBA* c.-932A>G  rs9932581 | A: FAM-GGCAGTAATGCTGGT-BHQ1 | 50 nM | 95 °C for 10 min  95 °C for 30 s  56 °C for 1 min  repeat 60 times |
|  | G: HEX-GGCAGCAATGCTGGT-BHQ1 | 50 nM |  |
|  | F: 5’-CTGGAATGGTGGCAGGAGT-3’ | 0.5µM |  |
|  | R: 5’- CGGGATGCTGGTTTACGAA-3’ | 0.5µM |  |
| **PCR/RFLP method** | | | |
| *CYBA* c.214C>T  rs4673 | F: 5’-TGCTTGTGGGTAAACCAAGG-3’ | 0.5µM | 95°C for 5 min  95 °C for 30 s  55 °C for 30 s  72 °C for 30 s  repeat 60 times  72 °C for 10 min |
|  | R: 5’-CACTTACCTCAGTGTTTTTCC-3’ | 0.5µM |  |
|  | dNTPs | 800 µM |  |
|  | 10x conc. PCR buffer | 2.0 µl |  |
|  | MgCl_2_ | 1.5 µM |  |
|  | Taq DNA polymerase | 0.25 U |  |
|  | Genomic DNA | 2.0 µl |  |
|  | PCR product of 353 bp digested with a restriction enzyme *Rsa*I which resulted in fragments:  T allele: 160 bp and 193 bp  C allele: 353 bp (non-digested) |  | 37°C for 1 h |

**Table S1.** Genotyping conditions. Primers, probes sequences.
